# Quantification of Ergot Alkaloids via Lysergic Acid Hydrazide—Development and Comparison of a Sum Parameter Screening Method

**DOI:** 10.3390/molecules28093701

**Published:** 2023-04-25

**Authors:** Maximilian Kuner, Jan Lisec, Tatjana Mauch, Jörg Konetzki, Hajo Haase, Matthias Koch

**Affiliations:** 1 Department of Analytical Chemistry and Reference Materials, Bundesanstalt für Materialforschung und-prüfung (BAM), Richard-Willstätter-Straße 11, 12489 Berlin, Germany; maximilian.kuner@bam.de (M.K.); jan.lisec@bam.de (J.L.); tatjana.mauch@bam.de (T.M.); 2 Institut Kirchhoff Berlin GmbH, Oudenarder Str. 16, 13347 Berlin, Germany; joerg.konetzki@mxns.com; 3 Institute of Food Technology and Food Chemistry, Technical University of Berlin, 10623 Berlin, Germany; haase@tu-berlin.de

**Keywords:** ergot alkaloids, mycotoxins, sum parameter, derivatization, hydrazinolysis

## Abstract

Ergot alkaloids are a group of mycotoxins occurring in products derived from various grasses (e.g., rye) and have been regulated in the EU recently. The new maximum levels refer to the sum of the six most common ergot alkaloids in their two stereoisomeric forms in different food matrices. Typically, these twelve compounds are individually quantified via HPLC-MS/MS or -FLD and subsequently summed up to evaluate food safety in a time-consuming process. Since all these structures share the same ergoline backbone, we developed a novel sum parameter method (SPM) targeting all ergot alkaloids simultaneously via lysergic acid hydrazide. After extraction and clean-up, in analogy to the current European standard method EN 17425 (ESM) for ergot alkaloid quantitation, the samples were derivatized by an optimized hydrazinolysis protocol, which allowed quantitative conversion after 20 min at 100 °C. The new SPM was evaluated against another established HPLC-FLD-based method (LFGB) and the HPLC-MS/MS-based ESM using six naturally contaminated rye and wheat matrix reference materials. While the SPM provided comparable values to the ESM, LFGB showed deviating results. Determined recovery rates, limits of detection and quantification of all three employed methods confirm that the new SPM is a promising alternative to the classical approaches for ergot alkaloid screening in food.

## 1. Introduction

Mycotoxins—toxic compounds formed by fungi in food and feed—have been a menace to mankind since the beginning of human sedentism and the associated agricultural crop cultivation and storage [1]. Among the various mycotoxins, ergot alkaloids play a special role, as they have been responsible for several thousands of death cases in the Middle Ages [2,3,4]. Even though the danger has been known since the 17th century [1], mass poisonings with ergot alkaloids in Europe were reported until the middle of the 20th century [5]. Ergotism, the disease resulting from the continuous consumption of ergot-alkaloid-contaminated products, can cause peripheral ischemia that might lead to the loss of limbs, elicit neuropathological manifestations, and, ultimately, result in the death of the sufferers. However, ergot alkaloids and their semi-synthetic derivatives are used in pharmaceutical applications [6], leading to an estimated production at a multiple tons per year scale [7].

Ergot alkaloids are formed as secondary metabolites by fungi of the *Claviceps* genus, with *Claviceps purpurea* being the most widely occurring species in Europe and Northern America [8,9]. Sclerotia—the wintering body of the fungus, containing the toxic alkaloids—grow on infested grasses, e.g., rye, wheat or barley. In the mill, the sclerotia are ground with the grain, spreading the ergot alkaloids within the flour [1].

**Figure 1:**
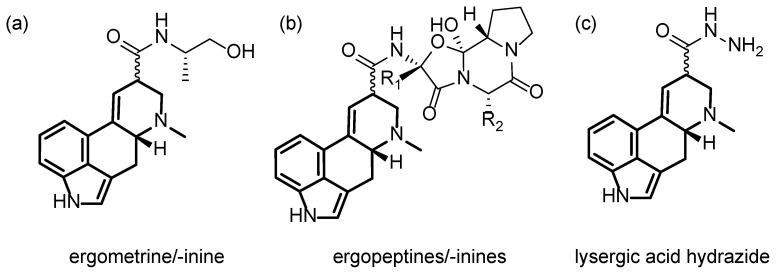
(**a**,**b**) Structure of the 12 major ergot alkaloids (ergoline structure highlighted): (**a**) ergometrine/-inine (*n* = 2), (**b**) ergopeptines/-inines: ergotamine/-inine, ergosine/-inine, ergocristine/-inine, ergocryptine/-inine and ergocornine/-inine with varying substituents R_1_/R_2_ (*n* = 10), (**c**) lysergic acid hydrazide as derivatization product for the sum parameter method.

Chemically, the group of ergot alkaloids is defined by one common feature: the ergoline backbone, a tetracyclic ring system derived from an indole moiety. Based on this framework, many natural and chemically modified derivatives exist, which are subdivided into four main classes: clavine-type alkaloids, simple lysergic acid derivatives, ergopeptines, and ergopeptams [10,11,12]. The 12 most common ergot alkaloids, which are usually quantified to determine the ergot alkaloid content of food samples, belong to the group of simple lysergic acid derivatives (Figure 1a) and ergopeptines (Figure 1b). The only difference between these major ergot alkaloids is their substituent and their stereoisomeric constitution at the C8 position of the ergoline system. The conformation at this atom, where the six different substituents of the major ergot alkaloids are attached, is important because just the *R*-isomers are biologically active. Nonetheless, the inactive *S*-conformation can isomerize, e.g., under acidic or basic conditions to the active form, and therefore, also needs to be quantified [11,13].

At the beginning of 2022, maximum levels for the ergot alkaloid content in different food matrices became effective in the EU (Table 1) [14]. The new regulation sets maximum contents referring to the sum of all twelve major ergot alkaloids, in contrast to the former regulatory limits defining just the sclerotia content in whole grain. 

To date, many methods for the quantification of ergot alkaloids have been reported and summarized in several review articles. Most of those methods are HPLC-based, detecting the major ergot alkaloids individually either via tandem mass spectrometry (MS/MS) or fluorescence detection (FLD) [13,15,16,17,18,19,20]. 

With the current European legislation not differentiating between the different compounds and referring just to the sum of all major ergot alkaloids, sum parameter methods (SPMs) seem much more appealing. However, just a few of those methods, targeting all ergot alkaloids at once, have been published, all of them suffering either from the heterogeneity of the ergot alkaloids (antibody-based methods) [20,21,22,23], from cross-reactivity against other indole-containing compounds [24,25] or from the use of problematic reagents [26].

The heterogeneity issue of the ergot alkaloids can be overcome by derivatization to a lysergic acid derivative as one uniform structure shared by all major ergot alkaloids. Previously, we reported a study examining and optimizing different cleaving methods regarding the suitability for a routine analysis SPM [27]. Hydrazinolysis of the ergot alkaloids to lysergic acid hydrazide (Figure 1c) turned out to be the most promising method in terms of handling, parallel sample work-up, and reaction time.

In this work, an analytical method for the quantification of ergot alkaloids via lysergic acid hydrazide as a sum parameter was developed, and the obtained results were compared with established approaches for ergot alkaloid quantitation. For comparison, we used the current HPLC-MS/MS-based European standard method described in EN 17425:2021 [28] (ESM) as well as the prior official HPLC-FLD-based method according to the German Food and Feed Code (LFGB) [29] as established and validated methods. Because certified reference materials for ergot alkaloids are not available, six naturally contaminated matrix reference materials were prepared and used for method validation in a two-laboratory comparison study.

## 2. Results

### 2.1. Derivatization and Synthesis of a Calibration Standard

Based on our pre-study comparing different cleaving methods, hydrazinolysis proved to be the best method for a routine analysis method [27]. However, most tests and optimizations in the previous publication were performed on the structurally similar dihydroergocristine (DHEC) as a model compound, differing just by saturation of one double bond (C9–C10 of the ergoline structure). As a proof of concept for the native ergot alkaloids, a mixture of the twelve major ergot alkaloids was treated with the optimized hydrazinolysis protocol. The chromatograms before and after hydrazinolysis were compared, showing that all ergopeptines reacted, and just the signals of ergometrine/-ine and the desired cleaving product remained [27].

Still, yield determination of the hydrazinolysis of native ergot alkaloids needed to be conducted. Hence, lysergic acid hydrazide was synthesized to be used not just for yield determination but also as a calibration standard in the final SPM. Lysergic acid hydrazide was produced by the reaction of ergotamine tartrate with hydrazine hydrate. The purity of both obtained hydrazide isomers (separated via preparative LC) was assigned by quantitative-NMR (*q*-NMR).

The synthesized compounds were used as calibrants in an HPLC-FLD method to determine the hydrazinolysis yield of ergotamine (tartrate) as a native ergot alkaloid. Hydrazinolysis of ergotamine was performed according to our previously published protocol [27]. To our surprise, the maximum yield for this reaction was just about 65% after 40 min (Figure S1 in Supplementary Materials). In contrast to the hydrazinolysis of DHEC, the yield decreased further with ongoing reaction time when cleaving the native ergot alkaloid.

**Figure 2:**
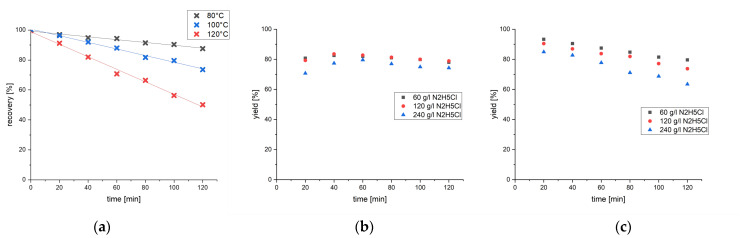
(**a**) Thermal stability test of lysergic acid hydrazide at 120, 100, and 80 °C in hydrazine hydrate in a thermoshaker. Recoveries were measured with HPLC-FLD (λ_ex_ = 330 nm, λ_em_ = 415 nm). (**b**,**c**) Hydrazinolysis of the twelve major ergot alkaloids with addition of varying amounts of hydrazine chloride at (**b**) 80 °C and (**c**) 100 °C. Yields were measured with HPLC-FLD (λ_ex_ = 330 nm, λ_em_ = 415 nm).

Degradation of the formed hydrazide was assumed as causal for the observed decrease in yield. To check this hypothesis, the stability of the hydrazide under reaction conditions was tested. To this end, the hydrazide was dissolved in the reaction mixture and agitated at various temperatures for two hours. Samples were taken every 20 min, and the recovery was measured by HPLC-FLD (Figure 2a). All applied temperatures resulted in a linear decrease in the hydrazide concentration. While at 120 °C, nearly half of the hydrazide was lost after two hours, lowering the temperature to 80 °C led to recoveries of about 90% after the same time. The linear decline and the high-temperature dependence underscore the hypothesis that the hydrazide degrades thermally. Moreover, the obtained solutions, after two hours of heating, were analyzed by HPLC-FLD, -DAD and -HRMS. None of the detectors allowed to determine a possibly formed side product.

However, reducing the temperature to prevent thermal decomposition did not solve the problem; maximum yields at 100 °C or 80 °C were about 70% after 40–60 min (Figure S2 in Supplementary Materials), as the formation reaction is also much slower at reduced temperatures.

A massive yield increase was achieved by replacing ammonium iodide (from our pre-study method) with hydrazinium chloride, which has been successfully employed in hydrazinolysis for peptide sequencing [30]. Full conversion of ergotamine tartrate to the corresponding hydrazide was reached after 20 min at 80 °C (Figure S3 in Supplementary Materials).

Owing to the much higher reactivity, the hydrazinolysis of ergometrine, which before remained unaffected, seemed possible. Thus, the hydrazinolysis of ergometrine under addition of equimolar amounts of hydrazinium chloride, bromide, or iodide was tested at 80 and 100 °C. About 60% maximum yield was achieved for the cleavage of ergometrine (Figure S4 in Supplementary Materials). Varying the anion of the hydrazinium salt had no significant influence on the reaction.

Finally, the cleavage of all twelve major ergot alkaloids was tested (Figure 2b,c). At 100 °C (Figure 2c), 93% maximum yield was achieved after 20 min. While the reaction at a lower temperature reached its peak 20 min later (Figure 2b), both curves show decomposition of the product afterwards. Hence, to prevent analyte losses, heating of the reaction must be stopped strictly thereafter. Because the yields decreased when increasing the amount of added hydrazine chloride too much, lower concentrations of hydrazine salt were tested (Table S1 in Supplementary Materials). Whereas the yields declined at both ends of the concentration range, an optimum was found at 30 g/L (3%) hydrazinium chloride in hydrazine hydrate.

**Figure 3:**
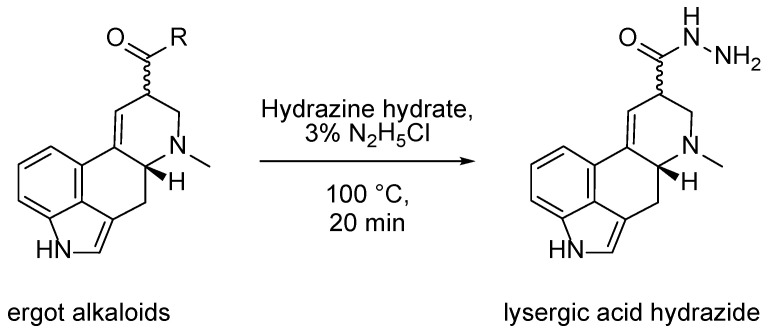
Hydrazinolysis used for derivatization of the ergot alkaloids to lysergic acid hydrazide for the determination of ergot alkaloids as a sum parameter.

In conclusion, reaction conditions of 100 °C for 20 min under addition of 3% hydrazine chloride in hydrazine hydrate solution were determined as the optimal derivatization parameters (Figure 3).

### 2.2. Sample Preparation

Prior to derivatization, several sample preparation steps are required in an analysis procedure. As grinding of the sample is not necessary in the case of flour samples, extraction is the first crucial step of the analysis. With the numerous methods published in the past, also many different extractions were described [13,15,17,20,31].

Since the solvent mixture must be removed before derivatization, extraction with more volatile solvents is beneficial, minimizing the evaporation time. Thus, extraction mixtures with lower aqueous content were considered preferable. 

**Figure 4:**
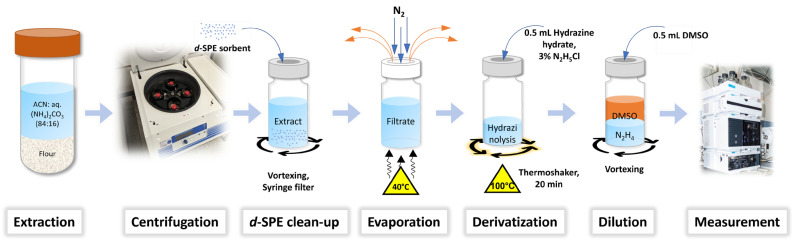
Workflow for the determination of ergot alkaloids via lysergic acid hydrazide as sum parameter method (SPM).

Therefore, samples were prepared and extracted in analogy to the current ESM for ergot alkaloid quantitation using 84% acetonitrile [28]. Additionally, this enables better comparability between the SPM and the established methods. As shown in Figure 4, the analysis procedure comprises extraction using acetonitrile: 0.02% aqueous ammonium carbonate solution (84:16) with subsequent centrifugation and dispersive solid-phase extraction (*d*-SPE) clean-up. The eluate obtained after removal of the *d*-SPE sorbent was dried in a nitrogen stream, and the derivatization mixture was added to the residue in the HPLC vial.

After hydrazinolysis, the mixture was diluted with dimethyl sulfoxide (DMSO) to avoid precipitation of the analyte upon cooling of the reaction mixture. Owing to its high boiling point, removal of the hydrazine hydrate after derivatization would be too time-consuming and thus not suitable for a quick SPM. Furthermore, reconstituting the residue in a common acetonitrile–aqueous solvent mixture (solvent for a sample is typically related to the HPLC-eluent composition) is unfavorable due to phase separation caused by the remaining hydrazine salt in the vial.

### 2.3. Measurement and Data Evaluation

After preparation, the samples were measured with HPLC-FLD. Due to the basic conditions caused by the remaining hydrazine hydrate in the vial, conversion between both stereoisomeric forms of lysergic acid hydrazide occurs. As both isomers are formed in the derivatization process anyway (also, the ergot alkaloids come in both isomeric forms), this does not affect the validity of the method. However, the calibrants are also dissolved in the same hydrazine hydrate–DMSO mixture as the samples and thus also isomerize. To target that problem, the areas of both isomer signals were summed up and just the sum was used for further calculations. This is valid because the FLD-response of both isomers is the same, which was shown by equal calibration curve slopes of both isomers in a solvent mixture without occurring isomerization (Figure S5 in Supplementary Materials). For further simplification, coelution of both isomers was attempted extensively. However, it remained unsuccessful even though numerous combinations of stationary phases, column sizes, or eluent compositions were tested.

### 2.4. Method Comparison Study

To enable comparison between the new SPM and the conventional methods, six matrix reference materials were produced because certified reference materials were not commercially available. Sclerotia were ground (<250 µm), mixed with blank rye and wheat flour, and homogenized to obtain materials with ~50 (L1), 150 (L2) and 250 µg/kg (L3) major ergot alkaloid content.

The novel SPM was compared to the HPLC-MS/MS-based current ESM for ergot alkaloid quantitation [28] and the former official German HPLC-FLD-based method (LFGB) [29]. 

These three methods were applied to each of the six reference materials in four replicate experiments by two participants (Lab 1/2). Due to the significant molar mass differences between the ergot alkaloids and lysergic acid hydrazide (about 1:2 for lysergic acid hydrazide to the ergopeptines), a direct comparison between the measured mass fractions is not helpful. Thus, the values were transferred to molar fractions to allow data comparison.

**Figure 5:**
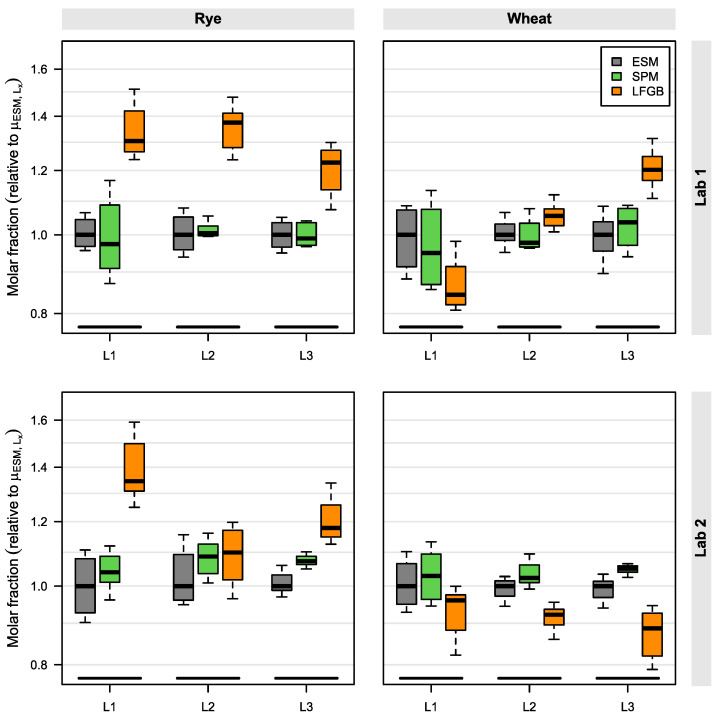
Comparison study for ergot alkaloid quantification in 6 flour samples (rye, wheat) including the new sum parameter method (SPM), the European standard method EN 17425 (ESM) and the German LFGB method. Relative molar fractions are normalized to the median obtained by the ESM of each material (rye/wheat), contamination level (L1 ~50 µg/kg; L2 ~150 µg/kg; L3 ~250 µg/kg major ergot alkaloid content) and participant (Lab 1/2) of the comparison study.

The results of the method comparison are depicted in Figure 5. The ESM was used as the reference method. For each material (rye/wheat), contamination level (L1–3), and participant (Lab 1/2), the values were normalized to the corresponding ESM median. This way, the normalized box plots allow a direct comparison between the different methods and contamination levels (in contrast to absolute values). The absolute molar ergot alkaloid contents of both participants are listed in the Supplementary Information (Tables S5–S10).

Overall, the SPM data are in good accordance with the ESM reference values. In the rye samples, the median values of the SPM compared to the ESM are between 97 to 102% for Lab 1 and between 104 to 109% for Lab 2. The trend that Lab 2 measured higher SPM values is also confirmed for the wheat samples, with SPM medians of 95 to 105% for Lab 1 and 103 to 106% for Lab 2 (referring to the respective ESM value). Even when considering all obtained values of both participants, the measured SPM data were within 87 to 118% of the reference value.

In contrast, the LFGB method showed much higher deviations from the reference method. Especially in rye, increased values were obtained for both participants, with relative median values to the ESM from 110 to 138%. In wheat, lower molar fractions were measured by LFGB: medians ranged from 84 to 120% for Lab 1 and 88 to 97% for Lab 2. Such differences between rye and wheat were not observed with the SPM.

In terms of method variances, all relative standard deviations (RSDs) were in the same low range. For the rye materials, the mean RSD over all contamination levels and participants was 5% for the ESM, 4% for the SPM, and 7% for LFGB. The same analysis for the wheat samples led to mean RSDs of 5% for all tested methods.

Limits of detection (LOD) and quantification (LOQ) were determined by the calibration curve method according to DIN 32645-2008. Analytical limits for the FLD-based methods (SPM, LFGB) were in the low ppb range. Whereas the LOQ for the new SPM was 0.655 µg/kg (LOD 0.185 µg/kg), the LOQs for LFGB varied from 0.251 to 1.530 µg/kg for the twelve major ergot alkaloids. Analytical limits in the low ppt area were achieved for the MS/MS-based ESM with LOQs ranging from 9.15 to 28.43 ng/kg, varying between the ergot alkaloids (Table S11 in Supplementary Materials).

**Figure 6:**
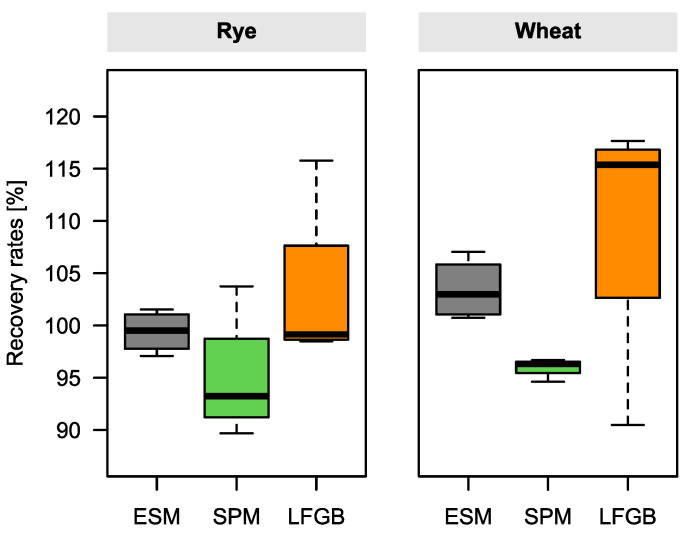
Determined ergot alkaloid recovery rates of EN 17425 (ESM), novel sum parameter method (SPM), and the German LFGB method in rye and wheat flour. Matrices were spiked with a mixed solution containing equimolar amounts of the 12 major ergot alkaloids. For ESM and LFGB, averaged recovery rates over all single ergot alkaloid recoveries for each replicate are depicted.

Recovery rates were determined by spiking blank rye and wheat samples with an ergot alkaloid mix solution containing equimolar amounts of each compound. Recovery rates of the official standard methods in Figure 6 were averaged over the individual values of the major ergot alkaloids in each replicate (*n* = 4) to simplify direct comparison to the SPM (recoveries for each ergot alkaloid in Figure S9 in Supplementary Materials). 

For all methods, slightly lower recoveries were observed in rye than in wheat. In rye flour, median recoveries of all three methods were in good accordance, ranging from 94 to 99%. In the wheat matrix, small differences between the methods were noticeable, leading to median recovery rates of 104% (ESM), 96% (SPM) and 115% (LFGB). The lowest recovery variances in both matrices were obtained with the ESM, followed by SPM and LFGB. 

Because the European maximum levels were set just recently, performance criteria for the determination of ergot alkaloids have not been defined yet. However, European Commission Regulation EC 401/2006 sets criteria for the determination of various other mycotoxins with typical recovery rates of 70–120%. The determined recovery rates of the three tested methods complied with that requirement.

## 3. Discussion

The crucial step of the SPM is transforming all ergot alkaloids into one uniform structure. Since the native ergot alkaloids showed lower temperature stability than the dihydro derivative used in our pre-study, hydrazinolysis conditions had to be further optimized. The addition of hydrazinium chloride led to remarkably increased yields and shortened reaction times. Due to the higher reactivity of the hydrazinium salt, cleavage of the much more stable ergometrine was achieved. This is a significant improvement to the only other published method trying to determine a sum parameter after derivatization of the ergot alkaloids [26], in which simple lysergic acid derivatives, such as ergometrine, remained unaffected. Further advantages of our novel SPM are the cheap and easier-to-handle reagents, the simple workflow allowing parallel derivatization of multiple samples, and the short 20 min reaction time [26].

A comparison study of the novel SPM with two official and validated standard methods (ESM, LFGB) was performed using six different reference materials. Their contamination levels were adjusted to be below or close to the current European maximum values for ergot alkaloids in wheat and rye. The comparison study showed that the novel SPM delivered similar values to the reference ESM for all tested contamination levels and flour types. This underscores that the typically quantified twelve compounds represent most of the ergot alkaloid contamination in food samples, as, by the derivatization, previously unconsidered ergot alkaloids would also be determined in the SPM.

However, contents measured with the LFGB method differed significantly from both other methods (ESM, SPM). Especially in rye, increased values were observed. A possible explanation are strong matrix interferences in the employed FLD, especially in the first minutes of the chromatogram when measuring rye samples. The overlapping matrix signals also explain the declining relative values to the ESM when measuring higher contaminated samples, as the influence of the matrix decreases with higher analyte peaks.

Regarding analytical parameters, the new SPM performed as well as the established methods. Recovery rates were between 89 to 105%. LOD (0.185 µg/kg) and LOQ (0.655 µg/kg) were in the low ppb range, hence, significantly below the current European maximum levels for ergot alkaloids. Still, the ESM provided the lowest limits, mainly due to the more sensitive MS/MS detection. Mean variances ranged from 4 to 7% RSD over all methods, materials, and participants, indicating the good performance of the methods.

While the SPM undoubtedly adds some steps in sample preparation, it overall has several advantages as a screening method compared to the classical approach. The costs for calibrants are substantially reduced since just one instead of twelve substances is required for analysis. This also facilitates chromatogram evaluation (twelve peaks vs. two summed-up peaks) and further calculations to determine the analyte content in the sample. The main advantage to the ESM is the use of HPLC-FLD instead of HPLC-MS/MS, resulting in much lower costs for instrumentation while—as shown in the comparison study—obtaining comparable results. Thus, even less well-equipped laboratories might perform such an analysis. In contrast to the tested FLD-based LFGB method, fewer matrix interferences were observed. Since just two instead of twelve substances are quantified, adapting the HPLC conditions to avoid matrix interferences is much easier.

Thus, the SPM represents a reliable and cost-reducing alternative to the classical approach for ergot alkaloid screening.

## 4. Materials and Methods

### 4.1. Chemicals and Equipment

All chemicals were used as purchased without further purification. Ergot alkaloid standards were obtained from RomerLabs Division Holding GmbH (Tulln, Austria). Ergotamine tartrate for derivatization tests, hydrazinium bromide, ammonium carbamate and the *q*-NMR-standard (1,2,4,5-tetrachloro-3-nitro-benzene, purity: 99.86%, Trace-Cert) were obtained from Sigma-Aldrich (St. Louis, MO, USA). DMSO, ethyl acetate, and acetonitrile (MS- and HPLC-grade) were obtained from Th. Geyer (Renningen, Germany). Ammonium carbonate and hydrazine hydrate (analytical reagent grade) were acquired from Fischer Scientific GmbH (Hampton, VS, USA). Bondesil PSA was purchased from Agilent Technologies Inc. (Waldbronn, Germany). Hydroiodic acid and 25% ammonia solution were obtained from Merck KGaA (Darmstadt, Germany). Hydrazinium chloride was purchased from abcr GmbH (Karlsruhe, Germany), and ammonium acetate was purchased from J.T. Baker (Deventer, The Netherlands). Basic alumina cartridges (Sep-Pak Alumina B, 50–300 µm, 1700 mg) were acquired from Waters Corp (Milford, MA, USA). All syringe filters were obtained from Macherey Nagel GmbH & Co. KG (Düren, Germany).

Hydrazinium iodide was produced by the reaction of hydrazine hydrate with hydroiodic acid and subsequent precipitation in *iso*-propanol.

For thermoshaking, an HLC MHR-13 thermoshaker (Hettich, Tübingen, Germany) was used. Compounds were freeze-dried in a Gamma 1-16 LSC plus freeze-drying system (Christ, Osterode, Germany). Centrifugation of the extracts was performed with a 6K15 centrifuge (Sigma, Osterode, Germany). HPLC vials were centrifuged in an RVC 2-25 CDplus speedvac system (Christ, Osterode, Germany).

Flours were purchased in a local supermarket, and the sclerotia were obtained from a grain breeding company in Lower Saxony, Germany.

Sclerotia were ground in a ZM 200 centrifugal mill (Retsch, Haan, Germany) and homogenized in a Reax 20 overhead shaker (Heidolph, Schwabach, Germany).

HPLC-FLD was measured with a 1290 infinity HPLC system, equipped with a G4226A autosampler, G1330B thermostat, G4204A quaternary pump, G1316C TCC, G4212B DAD detector, and a G1321B FLD detector (Agilent, Waldbronn, Germany).

Preparative HPLC was performed on a 1260 preparative system equipped with two G1361A preparative pumps, a G2260A preparative autosampler, a G1330B thermostat, a G1315D DAD detector, a G1364B fraction collecting system, and a 6130 quadrupole MS (Agilent, Waldbronn, Germany). A Phenomenex Luna Phenyl-Hexyl column (250 × 21.2 mm, 100 µm) was used.

HPLC-MS/MS data were measured on a 1290 infinity II HPLC system equipped with a G7167B autosampler, a G7120A pump, a G7116B thermostat, and a G6495C triple-quadrupole mass spectrometer (Agilent, Waldbronn, Germany).

High-resolution masses were measured with a 1290 infinity II system equipped with a G7167B autosampler, G7104A pump, G7116B MCT, G7117C DAD detector (Agilent, Waldbronn, Germany), and a TripleTOF 6600 mass spectrometer (Sciex, Darmstadt, Germany).

For the LFGB measurements and the hydrazinolysis tests, a Phenomenex Luna Phenyl-Hexyl column (250 × 4.6 mm, 5 µm) was used. SPM samples were measured with a Phenomenex Luna Phenyl-Hexyl column (150 × 3 mm, 5 µm). The ESM measurements were performed with a Phenomenex Gemini NX-C18 column (150 × 2 mm, 5 µm).

^1^H-NMR-spectra were measured on a Mercury-400 BB spectrometer (Varian, Palo Alto, CA, USA) at 400 MHz, and ^13^C-NMR were measured at 100 MHz.

All samples and calibration curves were produced and evaluated gravimetrically.

### 4.2. Synthesis of Lysergic Acid Hydrazide

For the synthesis of lysergic acid hydrazide, ergotamine tartrate (0.668 g, 1.01 mmol) was added to stirring hydrazine hydrate (11.5 mL, 233 mmol, 230 eq) under nitrogen atmosphere in a Schlenk flask equipped with a reflux condenser and heated to 140 °C (oil bath) for 90 min. After cool-down of the reaction mixture, most of the volatile compounds were removed under reduced pressure. The remaining oil was diluted with *iso*-propanol and separated by preparative HPLC (0.02% aq. NH_4_Ac: ACN; 0–8.20 min: 70:30; 8.20–11 min: 40:60; 11-11.20 min: 40:60; 11.20-16 min: 70:30; 16–40 min 70:30; flow: 20 mL/min). Fractions containing one of the product isomers were combined separately, and solvents were removed under reduced pressure and subsequently freeze-dried. Both isomers of lysergic acid hydrazide ((5*R*,8*R*)-isomer (0.059 g, 0.21 mmol, 21%); (5*R*,8S*)*-isomer (0.047 g, 0.17 mmol, 15%)) were obtained as gray solids. Purities of both compounds were determined by *q*-NMR ((5*R*,8*R*)-isomer: 81.7%; (5*R*,8*S*)-isomer: 93.1%). Conformation of the obtained isomers was assigned by retention time in HPLC.

***m/z*** (**measured**) = 283.1558 (theoretical: 283.1553, δ = 1.6 ppm)

*(* **5*R*,8*R*** *)* **-isomer:**

**^1^H-NMR** (**400 MHz, DMSO-*d_6_***)**:** δ 10.68 (s, 1H, N*H*), 9.18 (s, 1H, N*H*), 7.20–7.17 (m, 1H, C*H*_ar_), 7.07–7.01 (m, 3H, C*H*_ar_), 6.30 (s, 1H, C*H*), 4.27 (s, 2H, N*H*_2_), 3.46 (dd, *J* = 14.5, 5.5 Hz, 1H, C*H*), 3.38 (dd, *J* = 9.7, 4.7 Hz, 1H, C*H*), 2.97 (dd, *J* = 11.0, 5.3 Hz, 2H, C*H*_2_), 2.61–2.54 (m, 2H, C*H*_2_), 2.44 (s, 3H, C*H_3_*).

**^13^C-NMR** (**101 MHz, DMSO-*d_6_***)**:** δ 172.25, 136.67, 133.55, 128.31, 126.45, 123.56, 122.72, 119.81, 111.53, 110.32, 109.53, 62.71, 53.99, 43.64, 41.40, 27.19.

*(* **5*R*,8*S*** *)* **-isomer:**

**^1^H-NMR** (**400 MHz, DMSO-*d_6_***)**:** δ 10.68 (s, 1H, N*H*), 8.87 (s, 1H, N*H*), 7.20–7.17 (m, 1H, C*H*_ar_), 7.07–7.01 (m, 3H, C*H*_ar_), 6.45 (d, *J* = 3.8 Hz, 1H, C*H*), 4.22 (s, 2H, N*H*_2_), 3.42 (dd, *J* = 14.5, 5.5 Hz, 1H, C*H*), 3.18–2.94 (m, 3H, C*H_2_*/C*H*), 2.64–2.52 (m, 2H, C*H*_2_), 2.46 (s, 3H, C*H*_3_).

**^13^C-NMR** (**101 MHz, DMSO-*d_6_***)**:** δ 171.77, 136.20, 133.78, 127.83, 125.97, 122.23, 119.32, 118.76, 111.05, 109.83, 109.05, 62.22, 53.50, 43.14, 40.95, 26.71.

### 4.3. Hydrazinolysis Optimization and Stability Test of Lysergic Acid Hydrazide

Stock solutions of the respective ergot alkaloid (e.g., ergotamine, ergometrine) or the mixture of the major ergot alkaloids in *iso*-propanol were prepared (c = ~0.5 µmol/g per ergot alkaloid). A total of 250 µL (200 mg) of the stock solution was transferred to 20 mL headspace vials, and the solvent was removed in a nitrogen stream at 40 °C. Hydrazine hydrate (5 mL) and the corresponding amount of the respective salt (hydrazinium chloride, bromide, iodide) were added. Subsequently, the vials were shaken at 80, 100, or 120 °C in a thermoshaker, and samples (~0.1 mL, diluted with ~1.5 mL DMSO) were taken every 20 min for 2 h.

For the first hydrazinolysis yield determinations of ergotamine tartrate, ammonium iodide was added instead of a hydrazinium salt.

The measurement of the stability of lysergic acid hydrazide under reaction conditions was performed in the same manner as the optimization experiments described above. However, these tests were conducted without the addition of hydrazinium salts. 

All obtained samples were measured with HPLC-FLD (injection volume: 10 µL, HPLC-conditions: ACN: 0.02% aq. NH_4_Ac, 50:50, 0.8 mL/min, 20 min runtime; FLD-conditions: λ_ex_ = 330 nm, λ_em_ = 415 nm).

### 4.4. Sum Parameter Method (SPM) for Ergot Alkaloid Quantitation

A 100 mL-centrifugation tube was filled with 10 g flour sample and 50 mL extracting agent (ACN: 0.02% aq. (NH_4_)_2_CO_3_ (84:16)). After 30 min extraction at medium speed in an orbital shaker, the tubes were centrifuged (3000 rpm, 2900× *g*, 5 min). An aliquot (1.5 mL) of the supernatant was added to 75 mg of Bondesil PSA (40 µm) in a 4 mL amber vial and vortexed for 45 s. Subsequently, the slurry was transferred to a syringe and filtered through a syringe filter (PTFE, 25 mm, 0.2 µm) into an amber HPLC vial. In the next step, all volatile compounds were removed by a nitrogen stream (40 °C), and 0.5 mL derivatization mixture (3% hydrazinium chloride in hydrazine hydrate) was added. After crimping the vials, the samples were shaken for 20 min in a thermoshaker (100 °C, 1100 rpm) and then quickly removed to cool down to prevent analyte decomposition. Reaction in the thermoshaker allows the derivatization of 24 samples parallelly and thus enables an efficient and time-saving sample work-up. Samples were diluted with DMSO (0.5 mL) subsequently and vortexed thoroughly. Prior to HPLC measurement (Table 2), all vials were centrifuged to remove any remaining suspended matrix particles.

For the quantification by SPM, a six-point calibration was used, ranging from 1 µg/kg to 75 µg/kg (calibration solutions produced gravimetrically).

### 4.5. European Standard Method (ESM) for Ergot Alkaloid Quantitation

Sample extraction and clean-up were performed as described in Section 4.4. After *d*-SPE and syringe-filtration, the filtrates were directly measured by HPLC-MS/MS. As this is a well-described method, the employed HPLC and MS/MS parameters are moved to the Supplementary Information (Tables S2–S4).

### 4.6. LFGB-Method for Ergot Alkaloid Quantitation

A total of 50 mL eluent (ethyl acetate:methanol:aq. NH_3_, 75:5:7) was added to 10 g flour sample in a 100 mL centrifugation tube. Extraction was performed at medium speed for 45 min in an orbital shaker. Afterward, samples were centrifuged at 3000 rpm/2900× *g* for 10 min. In the next step, 5 mL of the supernatant was cleaned up over a basic aluminum oxide cartridge. An aliquot of 2 mL extract was then transferred to an amber vial. The volatile compounds were removed in a nitrogen stream (45 °C) and then reconstituted in 2 mL HPLC-eluent (ACN:0.02% aq. NH_4_COONH_2_ 1:1) by ultrasonication (15 min). After thorough vortexing and filtration through a syringe filter (PVDF, 25 mm, 0.45 µm), the samples were measured by HPLC-FLD.

HPLC conditions were as follows: injection volume: 20 µL; eluent: ACN: 0.02% aq. NH_4_NCOONH_2_ (50:50); flow-gradient: 0–17 min: 0.8 mL/min, 17–38 min: 1.5 mL/min; 38 min runtime; FLD conditions: λ_ex_ = 330 nm, λ_em_ = 415 nm

### 4.7. Determination of Recoveries and Analytical Limits (LOD/LOQ)

For the determination of the SPM, ESM, and LFGB recoveries, blank flour (rye/ wheat) was spiked with an ergot alkaloid mix solution containing equimolar amounts of each ergot alkaloid to obtain levels of ~17 nmol/kg (≙ ~10 µg/kg for ergopeptines and ~5 µg/kg for ergometrine, due to its much lower molecular weight) for each ergot alkaloid in the doped sample. Analysis was then performed as described above for the respective method, and the recoveries were calculated.

Analytical limits were determined by the calibration curve method according to DIN 32645-2008. Triplicates of ten equidistant calibration points over one order of magnitude were measured with a concentration range close to the noise of the respective detector. Subsequently, the obtained data were evaluated with the program DIN-Test to calculate the LOD and LOQ.

## Figures and Tables

**Table 1:** Maximum levels for the sum of the major ergot alkaloids in different matrices as set by Commission Regulation 2021/1399 [14].

	Matrix	Maximum Level
**Barley, wheat, spelt, oats**	Milling products (Ash content < 900 mg/100 g)	100 µg/kg 50 µg/kg (From July 2024)
	Milling products (Ash content ≥ 900 mg/100 g)	150 µg/kg
	Grains for final consumer	
**Rye**	Milling products	500 µg/kg 250 µg/kg (From July 2024)
	Grains for final consumer	
**Wheat**	Wheat gluten	400 µg/kg
	Processed-cereal-based food for infants	20 µg/kg

**Table 2:** SPM HPLC conditions: Phenomenex Luna Phenyl-Hexyl column (150 × 3 mm, 5 µm), column temperature: 40 °C, 10 µL injection, 15 min runtime, eluents: ACN, 0.02% aq. (NH_4_)_2_CO_3_ FLD parameters: λ_ex_ = 330 nm, λ_em_ = 415 nm.

Time (min)	Acetonitrile (%)	0.02% aq. (NH_4_)_2_CO_3_ (%)
0	10	90
1	10	90
8	90	10
9	90	10
10	10	90
15	10	90

## Data Availability

All relevant data are published in the article or the Supplementary Information.

## Supplementary Materials

The following supporting information can be downloaded at: https://www.mdpi.com/article/10.3390/molecules28093701/s1, Figures S1 and S2: Hydrazinolysis yields of ergotamine tartrate under addition of ammonium iodide at various temperatures. Figures S3 and S4: Hydrazinolysis yields of ergotamine (S3) or ergometrine (S4) under addition of different hydrazinium salts. Figure S5: Calibration curves of both lysergic acid hydrazide isomers in a solvent without occurring isomerization. Figures S6–S8: Exemplary HPLC-FLD chromatograms of the SPM calibration (S6), a rye (S7) and wheat (S8) sample. Figure S9: Recovery rates of the individual ergot alkaloids measured with ESM and LFGB. Figures S10 and S11: *q*-NMR spectra for the purity assessment of (5*R*,8*R*)- and (5*R*,8*S*)-lysergic acid hydrazide. Table S1: Hydrazinolysis yields of the twelve major ergot alkaloids, varying the amount of added hydrazinium chloride. Table S2: ESM HPLC parameters. Tables S3 and S4: ESM ion-source parameters and employed ESM MS/MS transitions. Tables S5 and S6: ESM-determined absolute molar contents in rye (S5) and wheat (S6). Tables S7 and S8: LFGB-determined absolute molar contents in rye (S7) and wheat (S8). Tables S9 and S10: SPM-determined absolute molar contents in rye (S9) and wheat (S10). Table S11: LODs and LOQs of ESM and LFGB.
