# Supplementary material for: Quantification of Ergot Alkaloids via Lysergic Acid Hydrazide—Development and Comparison of a Sum Parameter Screening Method
Source: Molecules. 2023 Apr 25;28(9):3701. doi: 10.3390/molecules28093701 (PMC10180493; doi:10.3390/molecules28093701)
Supplement: Supplementary file 1 [file molecules-28-03701-s001.zip › molecules-2367482-supplementary.pdf]

Quantification of ergot alkaloids via lysergic acid hydrazide – development and comparison of a new sum parameter screening method

Supplementary information

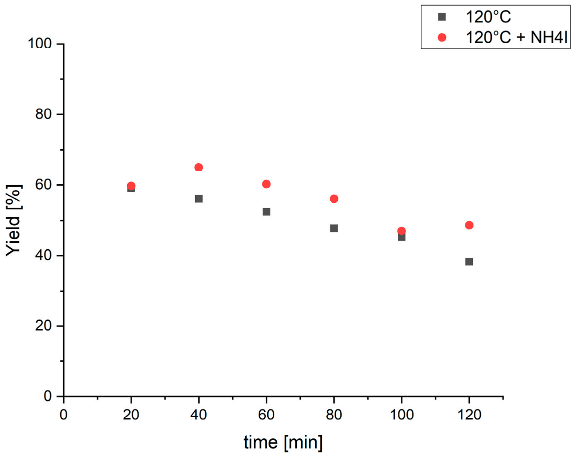

Figure S1: Hydrazinolysis yields of ergotamine tartrate with and without addition of ammonium iodide at 120 °C in a thermoshaker. Yields were measured with HPLC-FLD (λ_{ex}= 330 nm, λ_{em}= 415 nm).

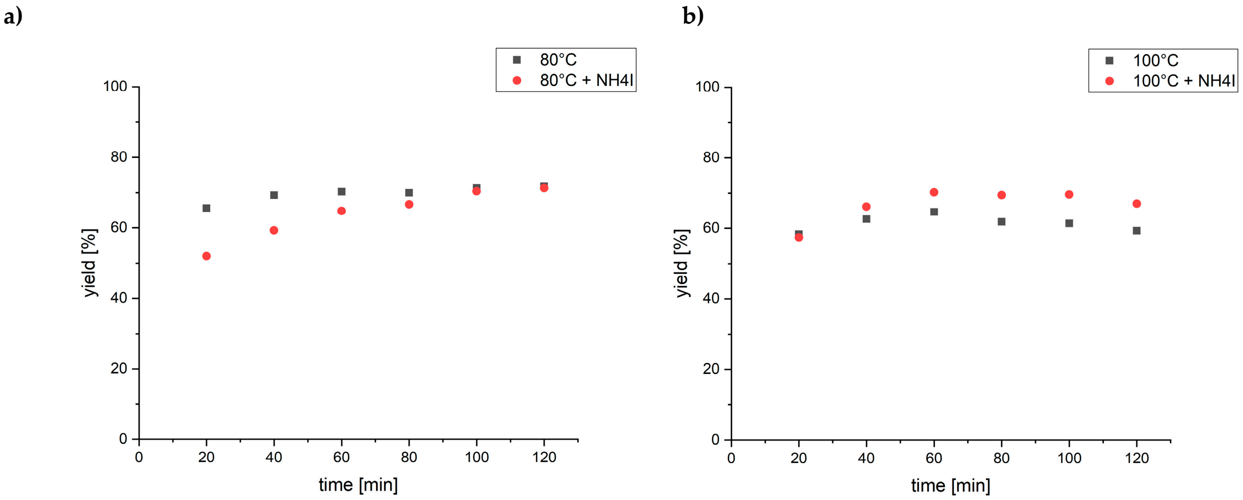

Figure S2: Hydrazinolysis yields of ergotamine tartrate with and without addition of ammonium iodide at either **a)** 80 °C or **b)** 100 °C in a thermoshaker. Yields were measured with HPLC-FLD (λ_{ex}= 330 nm, λ_{em}= 415 nm).

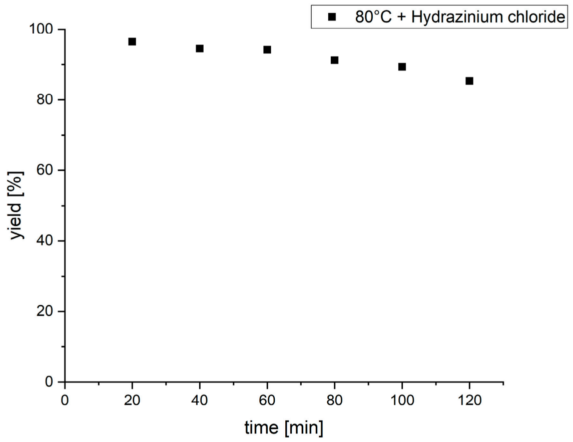

Figure S3: Hydrazinolysis yields of ergotamine tartrate with addition of hydrazinium chloride at 80 °C. Yields were measured with HPLC-FLD (λ_{ex}= 330 nm, λ_{em}= 415 nm).

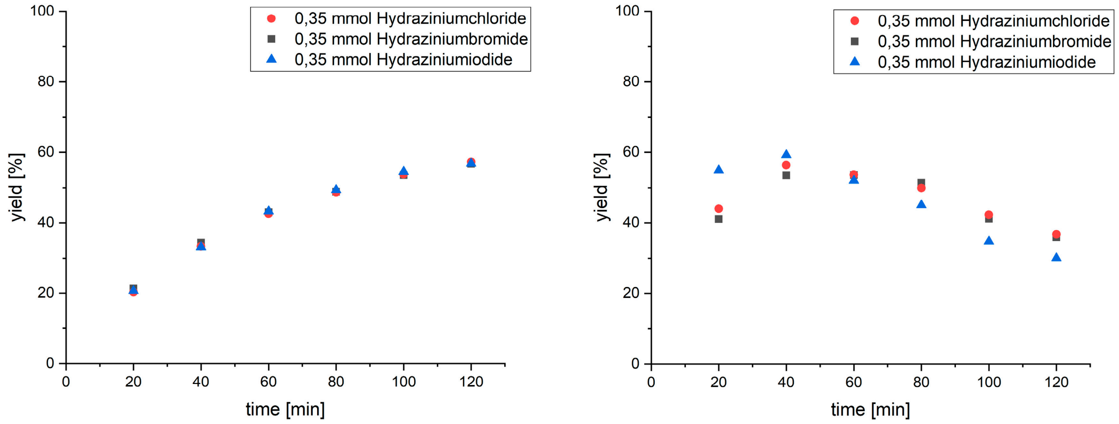

Figure S4: Hydrazinolysis of ergometrine under addition of different hydrazinium salts at either 80 °C (left) or 100 °C (right). Yields measured with HPLC-FLD (λ_{ex}= 330 nm, λ_{em}= 415 nm).

Table S1: Influence of the used amount of hydrazinium chloride on the hydrazinolysis of the twelve major ergot alkaloids after 20 minutes reaction time. Yields determined with HPLC-FLD (λ_{ex}= 330 nm, λ_{em}= 415 nm).

**Conc. N_2H_5Cl [g/l]**	**7.5**	**15**	**30**	**60**	**120**	**240**
**Yield [%]**	81	84	94	93	90	85

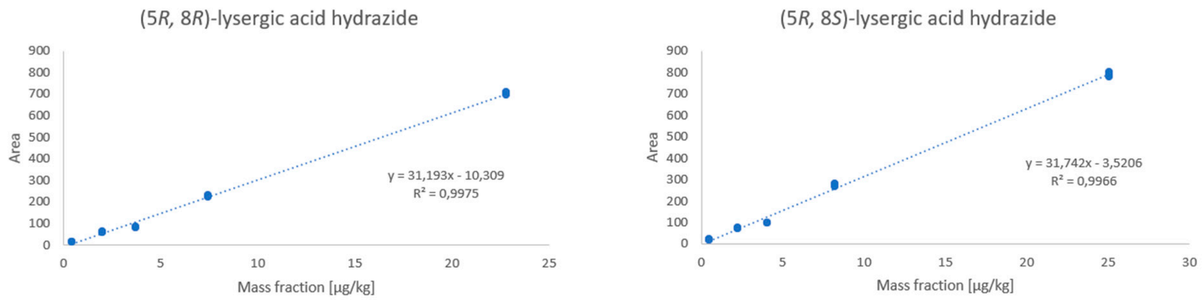

Figure S5 Calibration curves of both isomers of lysergic acid hydrazide in a solvent composition (ACN:0.02 % $(NH_4)_2CO_3$, 1:1) with low isomerization between both isomers measured with HPLC-FLD (λ_{ex}= 330 nm, λ_{em}= 415 nm). The similar slope of the calibration curves proofs the same response of both isomers in the FLD.

Table S2: Employed HPLC-conditions for the European standard-method (ESM) for ergot alkaloid quantitation of the major ergot alkaloids (EN 17425:2021): Phenomenex Gemini NX-C18 column (150 x 2 mm, 5 µm); column-temperature: 40 °C; 2 µL injection; 21 minutes runtime; flow: 0.5 mL/min; eluents: ACN, 0.02 % aq. (NH4)2CO3.

Time [min]	Acetonitrile [%]	0.02 % aq. $(NH_4)_2CO_3$ [%]
0	5	95
1	17	83
2	47	53
10	54	46
15	80	20
16	5	95
21	5	95

Table S3: Ion-source (ESI) parameters for the ESM-measurements.

Parameter	Value
Gas Temp [°C]	250
Gas Flow [L/min]	15
Nebulizer [psi]	20
SheathGasHeater [L/min]	350
SheathGasFlow [L/min]	12
Capillary [V]	2000

Table S4: MS/MS-transitions for the ESM-measurements.

Cpd Name	Prec Ion [M+H]+	Prod Ion [M+H]+	Dwell [ms]	Frag [V]	CE [V]	Cell Acc [V]
Ergometrine	326.19	223	20	166	28	4
Ergometrine	326.19	208	20	166	36	4
Ergometrinine	326.19	208	20	166	32	4
Ergometrinine	326.19	207	20	166	52	4
Ergosine	548.29	223	20	166	40	4
Ergosine	548.29	208	20	166	48	4
Ergosinine	548.29	530.3	20	166	16	4
Ergosinine	548.29	223	20	166	36	4
Ergotamine	582.27	564.3	20	166	16	4
Ergotamine	582.27	223	20	166	36	4
Ergotaminine	582.27	564.3	20	166	16	4
Ergotaminine	582.27	223	20	166	36	4
Ergocornine	562.31	268.1	20	166	28	4
Ergocornine	562.31	223	20	166	40	4
Ergocorninine	562.31	305.1	20	166	28	4
Ergocorninine	562.31	223	20	166	40	4
Ergocryptine	576.32	558.3	20	166	16	4
Ergocryptine	576.32	223	20	166	40	4
Ergocryptinine	576.32	558.3	20	166	16	4
Ergocryptinine	576.32	223	20	166	40	4
Ergocristine	610.31	592.3	20	166	16	4
Ergocristine	610.31	223	20	166	40	4
Ergocristinine	610.31	592.3	20	166	12	4
Ergocristinine	610.31	223	20	166	40	4

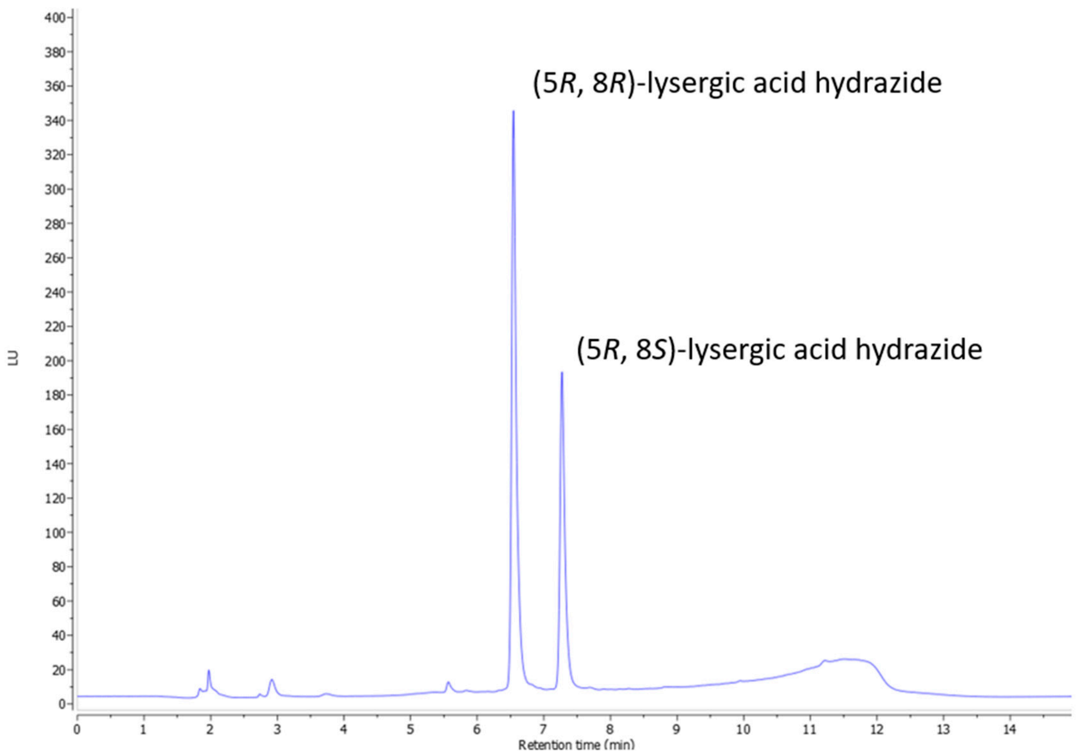

Figure S6: Exemplary HPLC-FLD chromatogram (λ_{ex}= 330 nm, λ_{em}= 415 nm) of the SPM calibration.

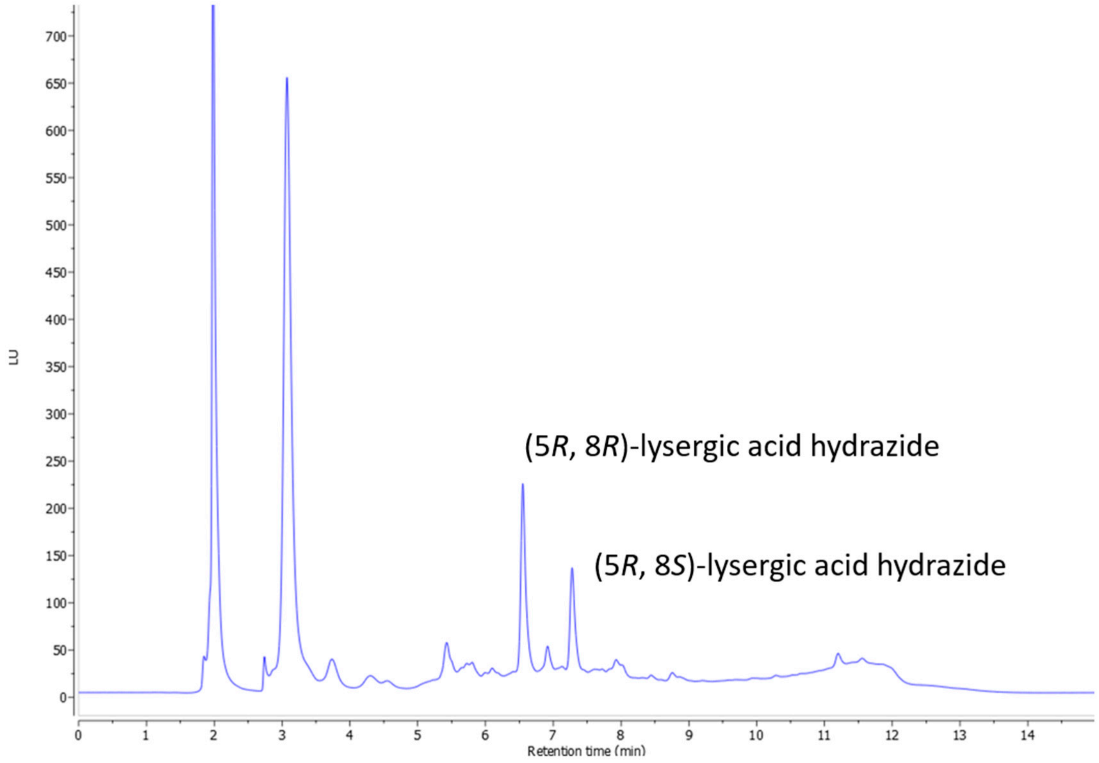

Figure S7: Exemplary HPLC-FLD chromatogram (λ_{ex}= 330 nm, λ_{em}= 415 nm) of a SPM rye sample.

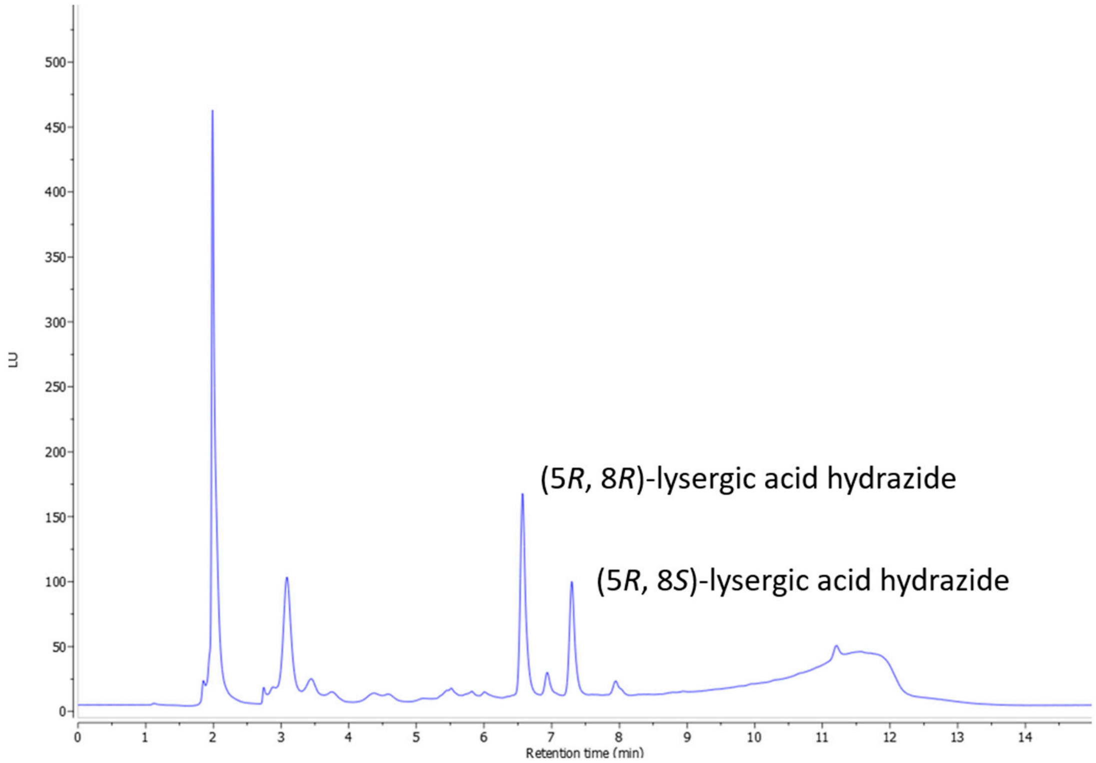

Figure S8: Exemplary HPLC-FLD chromatogram (λ_{ex}= 330 nm, λ_{em}= 415 nm) of a SPM wheat sample.

Table S5: Determined molar ergot alkaloid contents of the rye flour samples in nmol/kg, measured with the reference method EN 17425:2021 (ESM). Em: ergometrine, Es: ergosine, Ea: ergotamine, Eco: ergocornine, Ecp: ergocryptine, Ecs: ergocristine. The suffix -n stands for the corresponding -inine.

Participant	Sample	Em	Emn	Es	Ea	Eco	Ecp	Ecs	Esn	Ean	Econ	Ecpn	Ecsn	Sum	Avg. Cont.	Std. Dev	RSD
Lab 1	**R1-1**	13.2	5.0	11.4	16.6	11.2	8.8	20.0	6.1	17.6	4.6	6.3	6.9	127.6	121.3	4.9	4.0%
	**R1-2**	17.7	5.5	10.8	11.5	7.2	6.9	19.5	5.8	15.2	3.2	5.1	7.4	115.8			
	**R1-3**	15.0	5.1	11.3	13.2	8.1	7.2	19.6	6.2	14.8	3.7	5.6	7.5	117.4			
	**R1-4**	15.5	5.3	10.7	12.4	9.9	8.3	21.0	6.1	15.5	4.6	7.1	8.0	124.3			
	**R2-1**	36.3	9.2	29.3	31.7	20.1	16.7	48.3	15.6	37.4	9.2	13.2	17.4	284.6	279.8	13.8	4.9%
	**R2-2**	33.7	8.9	25.8	28.0	17.6	16.5	48.0	14.8	37.0	8.7	13.9	19.7	272.6			
	**R2-3**	33.4	8.9	24.9	25.6	18.2	16.3	44.2	14.3	35.9	8.9	14.2	17.7	262.5			
	**R2-4**	39.5	10.1	29.9	33.3	19.8	18.5	50.7	16.3	38.5	9.0	14.8	18.8	299.3			
	**R3-1**	58.0	13.6	44.6	49.5	31.5	30.0	82.9	24.5	56.1	14.8	23.8	30.8	460.2	479.0	16.7	3.5%
	**R3-2**	64.6	15.1	47.8	55.2	32.1	32.1	89.9	25.8	61.2	14.7	27.3	32.9	498.6			
	**R3-3**	58.0	13.5	47.5	52.8	29.1	26.8	85.4	25.6	59.3	13.6	22.0	31.1	464.8			
	**R3-4**	62.3	14.1	47.0	55.9	29.5	29.4	93.0	25.9	62.5	14.7	23.6	34.4	492.4			
Lab 2	**R1-1**	17.5	6.0	11.9	15.2	8.8	9.6	23.7	7.6	16.1	4.4	7.8	10.4	139.0	131.8	10.5	7.9%
	**R1-2**	13.7	5.1	11.4	15.8	7.9	7.1	18.0	7.2	15.7	3.9	6.1	7.8	119.6			
	**R1-3**	24.9	7.1	14.0	17.3	8.2	7.1	20.6	9.3	15.6	4.3	7.0	9.4	144.9			
	**R1-4**	15.2	5.2	9.7	16.5	7.8	7.6	19.7	6.6	15.9	4.1	6.7	8.8	123.7			
	**R2-1**	40.4	10.6	28.6	32.0	21.0	19.3	57.0	18.8	39.1	10.9	17.6	25.5	320.8	320.6	24.5	7.6%
	**R2-2**	36.2	9.4	31.6	31.0	21.0	18.0	44.5	21.4	34.0	10.7	17.3	21.3	296.5			
	**R2-3**	37.7	9.4	29.3	35.9	19.5	18.1	46.9	19.4	39.6	10.1	17.0	22.1	304.9			
	**R2-4**	42.1	10.5	30.7	44.4	24.2	21.8	56.9	21.7	44.7	13.1	21.0	29.3	360.3			
	**R3-1**	54.4	13.3	43.5	57.5	29.8	27.3	72.6	29.0	59.8	15.4	25.9	34.3	462.9	477.2	13.7	2.9%
	**R3-2**	60.6	14.7	43.0	49.1	28.4	25.8	76.4	31.8	61.7	16.5	28.3	41.9	478.2			
	**R3-3**	56.0	13.6	43.4	59.2	28.3	26.3	70.3	30.4	61.7	15.7	27.6	36.3	468.7			
	**R3-4**	57.3	14.3	46.3	62.7	29.4	30.1	68.2	34.4	69.3	17.7	31.1	38.1	498.8			

Table S6 Determined molar ergot alkaloid contents of the wheat flour samples in nmol/kg, measured with the reference method EN 17425:2021 (ESM). Em: ergometrine, Es: ergosine, Ea: ergotamine, Eco: ergocornine, Ecp: ergocryptine, Ecs: ergocristine. The suffix -n stands for the corresponding -inine.

Participant	Sample	Em	Emn	Es	Ea	Eco	Ecp	Ecs	Esn	Ean	Econ	Ecpn	Ecsn	Sum	Avg. Cont.	Std. Dev	RSD
Lab 1	W1-1	13.3	4.9	14.3	17.9	7.8	8.1	17.4	6.8	14.1	3.6	7.4	6.9	122.6	113.8	9.2	8.1%
	W1-2	16.5	4.8	10.5	11.2	7.6	6.8	15.8	5.1	9.9	3.5	5.0	5.8	102.3			
	W1-3	16.6	4.8	11.5	13.3	6.9	6.1	17.1	5.8	10.6	3.1	5.5	5.9	107.2			
	W1-4	17.3	5.0	15.8	16.0	7.3	7.2	19.7	6.9	11.9	3.4	5.6	6.8	123.0			
	W2-1	41.1	9.1	32.0	34.4	18.9	17.7	47.6	14.6	24.9	8.6	14.2	16.7	279.8	284.0	8.5	3.0%
	W2-2	37.9	8.8	38.1	41.1	21.9	18.8	47.8	17.0	26.9	9.1	13.7	16.4	297.7			
	W2-3	39.3	8.8	28.5	39.7	16.8	17.4	46.8	12.9	27.5	7.8	13.7	15.7	274.9			
	W2-4	39.9	9.0	31.8	38.5	19.2	18.4	46.5	14.9	27.2	8.9	13.6	16.0	283.8			
	W3-1	64.8	13.6	50.7	59.8	30.6	31.8	82.4	22.8	41.5	13.7	23.0	27.3	461.8	458.7	26.2	5.7%
	W3-2	61.3	12.7	47.0	57.1	25.6	26.2	72.9	21.5	39.7	12.0	19.5	24.5	420.2			
	W3-3	61.9	12.8	50.2	66.0	29.4	27.9	81.5	23.3	43.3	13.5	21.5	27.3	458.7			
	W3-4	69.7	14.3	53.1	68.9	30.5	32.9	88.3	24.0	46.3	14.2	22.9	29.0	494.1			
Lab 2	W1-1	11.9	4.3	10.7	19.8	6.8	6.7	16.9	5.1	8.7	3.1	4.6	5.1	103.7	111.5	6.8	6.1%
	W1-2	16.6	4.6	11.1	22.9	7.9	7.5	17.1	5.2	9.3	3.1	5.2	5.3	115.7			
	W1-3	14.7	4.4	9.3	19.3	8.1	7.3	16.0	4.6	8.6	3.5	5.1	5.2	106.1			
	W1-4	19.4	5.2	12.6	18.2	7.2	7.0	20.4	5.6	10.1	3.4	5.0	6.3	120.4			
	W2-1	42.0	8.9	36.7	42.6	21.3	18.6	64.4	15.7	20.6	8.3	12.6	18.1	309.9	303.3	7.2	2.4%
	W2-2	46.8	9.5	32.5	46.0	20.4	17.5	51.1	13.7	20.8	8.1	12.1	14.8	293.3			
	W2-3	43.6	9.2	29.7	63.7	17.6	18.3	55.1	12.8	24.4	7.5	12.4	15.9	310.2			
	W2-4	41.5	9.1	36.2	51.6	19.6	17.7	51.6	15.3	21.8	8.0	12.2	14.9	299.6			
	W3-1	69.9	13.2	51.2	78.6	29.6	29.0	86.4	20.7	34.5	11.9	19.5	23.5	468.0	490.9	14.7	3.0%
	W3-2	71.7	14.2	52.2	79.4	34.7	31.0	91.9	21.3	35.8	13.5	19.7	24.9	490.3			
	W3-3	68.7	13.4	54.0	82.6	34.8	32.0	94.2	21.7	35.1	13.8	21.0	26.0	497.4			
	W3-4	72.4	14.1	55.6	78.6	40.6	33.1	93.9	22.2	34.7	15.3	21.8	25.7	508.0			

Table S7: Determined molar ergot alkaloid contents of the rye flour samples in nmol/kg, measured with the LFGB-method. Em: ergometrine, Es: ergosine, Ea: ergotamine, Eco: ergocornine, Ecp: ergocryptine, Ecs: ergocristine. The suffix -n stands for the corresponding -inine.

Participant	Sample	Em	Emn	Es	Ea	Eco	Ecp	Ecs	Esn	Ean	Econ	Ecpn	Ecsn	Sum	Avg. Cont.	Std. Dev	RSD
Lab 1	R1-1	29.5	8.3	31.6	33.9	15.2	12.0	21.1	8.0	9.1	3.9	3.1	6.3	181.9	161.8	12.2	7.5%
	R1-2	24.1	7.9	27.3	33.0	13.3	9.4	19.3	6.2	9.2	3.2	2.2	5.9	161.0			
	R1-3	19.0	8.0	26.3	31.2	14.1	10.8	16.7	6.3	8.8	3.2	2.5	4.9	151.7			
	R1-4	24.0	8.1	25.4	30.3	12.4	10.2	17.3	5.8	8.4	2.8	2.6	5.2	152.6			
	R2-1	70.5	12.0	52.6	82.2	24.7	22.0	50.1	15.3	26.2	7.9	5.8	16.1	385.4	377.6	22.0	5.8%
	R2-2	61.2	11.9	45.6	68.5	28.0	22.2	43.8	12.9	21.9	8.8	6.4	13.6	344.8			
	R2-3	71.9	12.5	64.9	84.7	25.6	24.1	46.4	19.0	25.8	8.1	7.0	15.7	405.6			
	R2-4	70.3	11.8	53.4	70.9	26.9	25.5	48.4	13.9	23.1	8.2	7.2	15.0	374.6			
	R3-1	94.4	16.6	73.4	116.9	40.9	37.3	83.8	23.7	37.5	12.9	11.5	26.3	575.0	577.2	38.7	6.7%
	R3-2	82.3	15.0	64.6	104.2	36.6	36.4	75.4	20.7	33.4	11.6	10.5	24.4	515.2			
	R3-3	117.9	17.1	84.8	107.1	41.7	34.8	83.3	25.8	36.3	13.7	11.6	28.1	602.3			
	R3-4	111.1	17.8	82.5	119.0	41.2	35.8	85.6	26.2	40.8	14.5	12.3	29.2	616.0			
Lab 2	R1-1	22.8	5.4	35.9	40.6	18.7	12.5	22.2	12.4	15.9	5.5	4.5	11.5	207.9	183.0	15.3	8.4%
	R1-2	20.9	2.7	31.3	35.5	20.9	13.9	17.1	8.2	12.1	4.5	4.6	9.2	180.9			
	R1-3	19.4	3.7	28.9	36.0	18.4	12.7	18.9	8.2	13.7	4.5	3.7	8.6	176.8			
	R1-4	19.3	4.6	26.4	34.4	16.6	10.8	16.0	8.1	14.6	3.9	3.6	8.1	166.5			
	R2-1	53.8	10.0	42.4	66.4	28.5	25.6	49.6	17.5	33.4	10.6	9.9	24.7	372.5	341.1	26.1	7.6%
	R2-2	49.6	10.1	44.1	61.6	28.9	24.2	46.4	19.6	30.6	11.2	9.6	22.7	358.7			
	R2-3	39.3	9.7	40.8	55.8	27.0	21.8	45.5	17.3	29.4	10.4	8.3	21.9	327.1			
	R2-4	37.1	10.3	37.9	58.2	22.2	21.2	40.5	15.8	30.2	8.4	8.1	16.4	306.1			
	R3-1	84.1	18.5	65.9	113.1	49.2	43.4	87.3	32.3	60.5	19.3	16.7	39.7	630.0	569.7	36.4	6.4%
	R3-2	75.9	14.3	59.3	94.0	37.1	33.4	77.9	29.4	49.7	14.3	14.2	33.1	532.7			
	R3-3	85.2	14.9	63.8	92.9	42.7	37.8	79.4	31.2	48.5	16.5	15.2	34.0	562.0			
	R3-4	76.6	16.5	64.1	96.5	39.2	37.1	78.4	31.5	50.9	15.1	14.5	33.9	554.2			

Table S8: Determined molar ergot alkaloid contents of the wheat flour samples in nmol/kg, measured with the LFGB-method. Em: ergometrine, Es: ergosine, Ea: ergotamine, Eco: ergocornine, Ecp: ergocryptine, Ecs: ergocristine. The suffix -n stands for the corresponding -inine.

Participant	Sample	Em	Emn	Es	Ea	Eco	Ecp	Ecs	Esn	Ean	Econ	Ecpn	Ecsn	Sum	Avg. Cont.	Std. Dev	RSD
Lab 1	**W1-1**	14.1	3.5	11.9	20.5	6.0	5.9	13.1	4.1	6.8	3.0	2.0	4.6	95.4	99.5	6.6	6.6%
	**W1-2**	15.1	3.1	13.1	27.1	6.2	6.6	16.5	4.1	8.5	2.9	2.1	5.3	110.7			
	**W1-3**	14.2	4.0	11.4	19.8	6.4	6.2	12.7	3.7	6.7	2.6	2.1	4.3	94.1			
	**W1-4**	17.7	3.5	10.8	21.4	6.3	6.0	12.7	3.2	7.3	2.9	2.0	4.1	97.9			
	**W2-1**	45.6	6.7	34.1	62.5	18.6	20.4	43.8	11.6	19.9	7.5	6.8	14.9	292.5	298.1	7.9	2.7%
	**W2-2**	47.4	6.9	39.9	60.1	17.5	17.5	45.5	13.2	20.1	6.7	5.8	14.4	295.0			
	**W2-3**	48.8	7.6	35.1	61.3	19.1	19.1	42.0	11.4	21.0	7.4	6.5	13.6	292.9			
	**W2-4**	48.0	7.4	42.4	63.1	23.0	20.5	42.2	13.1	21.1	8.8	6.8	15.3	311.8			
	**W3-1**	95.7	10.1	75.9	130.0	35.5	33.1	82.3	27.1	47.2	15.0	12.3	31.0	595.1	556.3	27.7	5.0%
	**W3-2**	88.6	10.0	68.4	113.1	32.1	35.0	84.2	24.4	38.7	14.5	12.3	29.9	551.4			
	**W3-3**	99.7	11.8	64.8	117.6	32.3	36.0	83.4	21.3	40.0	14.2	11.9	28.3	561.2			
	**W3-4**	85.5	11.1	66.2	105.2	30.2	31.6	77.3	22.2	36.1	13.5	11.3	27.2	517.3			
Lab 2	**W1-1**	14.2	3.9	13.7	19.0	7.2	6.6	15.0	7.0	9.3	3.7	2.9	5.6	107.9	103.2	6.8	6.6%
	**W1-2**	12.7	3.1	10.6	13.0	6.5	5.8	13.2	5.4	8.4	3.5	2.6	6.7	91.5			
	**W1-3**	12.3	2.6	12.5	19.2	8.3	6.8	14.5	6.4	9.2	4.1	3.1	6.7	105.6			
	**W1-4**	12.1	2.4	10.4	20.9	6.8	5.8	19.3	7.1	10.4	3.1	2.3	7.1	107.6			
	**W2-1**	35.6	7.6	26.9	49.0	16.2	15.8	42.9	13.9	25.2	8.2	6.9	17.7	266.0	279.5	8.5	3.0%
	**W2-2**	41.3	9.1	30.9	49.5	18.6	16.1	42.8	16.7	24.6	9.0	7.5	17.1	283.3			
	**W2-3**	40.6	8.3	28.5	49.4	17.7	17.2	42.7	14.1	27.2	9.2	6.9	18.1	279.8			
	**W2-4**	35.8	8.3	28.5	60.1	18.5	15.9	42.6	14.0	31.7	8.1	7.3	18.0	289.0			
	**W3-1**	64.8	13.5	47.8	80.3	29.5	32.8	74.9	22.7	42.0	14.3	11.5	30.4	464.6	433.0	29.1	6.7%
	**W3-2**	49.9	11.4	38.0	71.5	24.2	27.9	61.9	18.5	38.7	11.4	10.7	26.7	390.8			
	**W3-3**	54.7	12.0	42.6	78.1	25.6	29.6	65.6	21.1	41.3	12.6	11.4	27.0	421.6			
	**W3-4**	60.7	14.4	47.7	80.4	29.0	32.1	67.8	24.2	42.8	14.1	12.8	29.1	455.1			

Table S9: Determined content of lysergic acid hydrazide in the tested rye-flour samples. Samples were measured by the novel sum-parameter method (SPM).

Participant	Sample	Lysergic acid hydrazide [nmol/kg]	Avg. Cont. [nmol/kg]	Std. Dev [nmol/kg]	RSD
Lab 1	**R1-1**	139.7	120.3	12.7	10.6%
	**R1-2**	105.4			
	**R1-3**	114.0			
	**R1-4**	122.2			
	**R2-1**	287.2	282.1	4.3	1.5%
	**R2-2**	278.4			
	**R2-3**	277.3			
	**R2-4**	285.5			
	**R3-1**	464.2	478.8	14.4	3.0%
	**R3-2**	490.3			
	**R3-3**	464.8			
	**R3-4**	495.7			
Lab 2	**R1-1**	143.8	137.3	6.0	4.3%
	**R1-2**	129.8			
	**R1-3**	142.3			
	**R1-4**	133.1			
	**R2-1**	343.0	338.3	16.4	4.8%
	**R2-2**	315.6			
	**R2-3**	333.6			
	**R2-4**	361.0			
	**R3-1**	499.2	508.3	7.6	1.5%
	**R3-2**	510.8			
	**R3-3**	503.8			
	**R3-4**	519.4			

Table S10: Determined content of lysergic acid hydrazide in the tested wheat-flour samples. Samples were measured by the novel sum-parameter method (SPM).

Participant	Sample	Lysergic acid hydrazide [nmol/kg]	Avg. Cont. [nmol/kg]	Std. Dev [nmol/kg]	RSD
Lab 1	**W1-1**	129.6	111.4	12.7	11.4%
	**W1-2**	98.6			
	**W1-3**	100.5			
	**W1-4**	116.9			
	**W2-1**	280.0	282.2	12.2	4.3%
	**W2-2**	302.6			
	**W2-3**	274.0			
	**W2-4**	272.0			
	**W3-1**	499.0	472.0	25.8	5.5%
	**W3-2**	434.5			
	**W3-3**	461.8			
	**W3-4**	492.7			
Lab 2	**W1-1**	104.9	114.1	7.8	6.8%
	**W1-2**	117.8			
	**W1-3**	108.9			
	**W1-4**	124.9			
	**W2-1**	334.1	315.9	11.0	3.5%
	**W2-2**	305.3			
	**W2-3**	310.2			
	**W2-4**	313.7			
	**W3-1**	517.0	518.7	5.6	1.1%
	**W3-2**	511.0			
	**W3-3**	520.1			
	**W3-4**	526.6			

Table S11: Limits of detection and quantification of the tested established methods (ESM, LFGB) determined by the calibration-curve method according to DIN 32645-2008.
Em: ergometrine, Es: ergosine, Ea: ergotamine, Eco: ergocornine, Ecp: ergocryptine, Ecs: ergocristine. The suffix -n stands for the corresponding -inine.

Method		Em	Emn	Es	Ea	Eco	Ecp	Ecs	Esn	Ean	Econ	Ecpn	Ecsn
**ESM**	**LOD [ng/kg]**	8.10	4.89	3.19	5.32	8.23	5.11	6.58	2.52	3.10	5.24	3.34	4.31
	**LOQ [ng/kg]**	28.00	17.4	11.50	18.84	28.43	18.15	23.02	9.15	11.22	18.56	12.02	15.39
**LFGB**	**LOD [µg/kg]**	0.179	0.068	0.267	0.253	0.449	0.268	0.250	0.197	0.370	0.178	0.502	0.243
	**LOQ [µg/kg]**	0.642	0.251	0.944	0.897	1.530	0.946	0.885	0.706	1.282	0.641	1.700	0.863

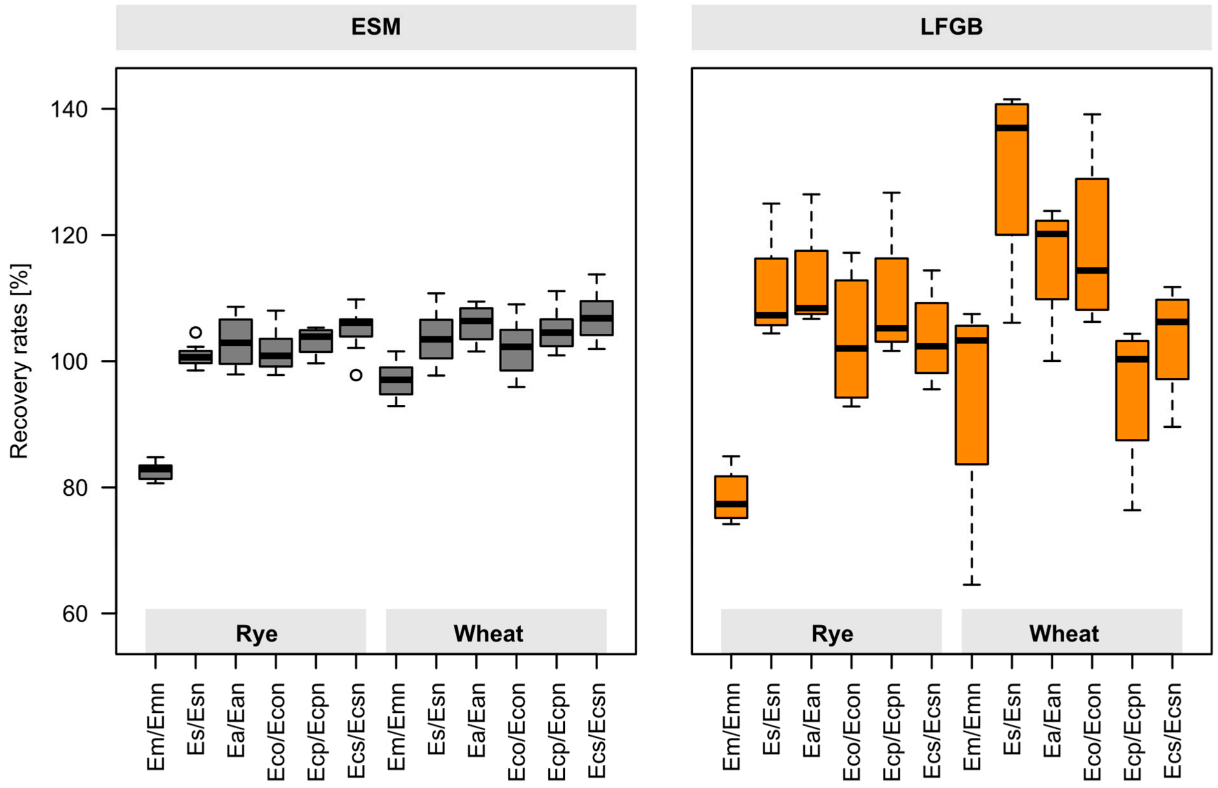

Figure S9: Recovery rates of the individual ergot alkaloids obtained by measurement of spiked blank rye and wheat flour either by ESM or LFGB-method. Em: ergometrine, Es: ergosine, Ea: ergotamine, Eco: ergocornine, Ecp: ergocryptine, Ecs: ergocristine. The suffix -n stands for the corresponding -inine.

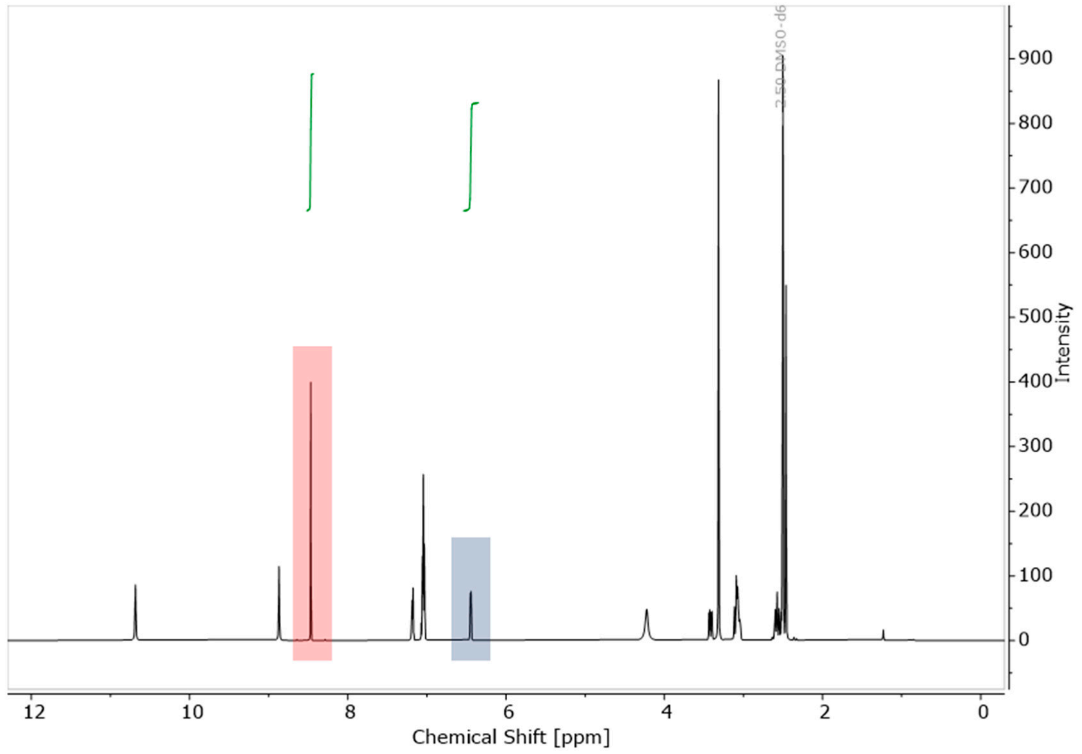

Figure S10: *q*-NMR-Spectrum for the purity assessment of (5*R*, 8*S*)-lysergic acid hydrazide. Red highlighted area at 8.47 ppm is the signal of the internal calibration standard 1,2,4,5-Tetrachloro-3-nitrobenzene. The blue area at 6.45 ppm is from the proton attached to the C9-position of the analyte, which was used for quantification.

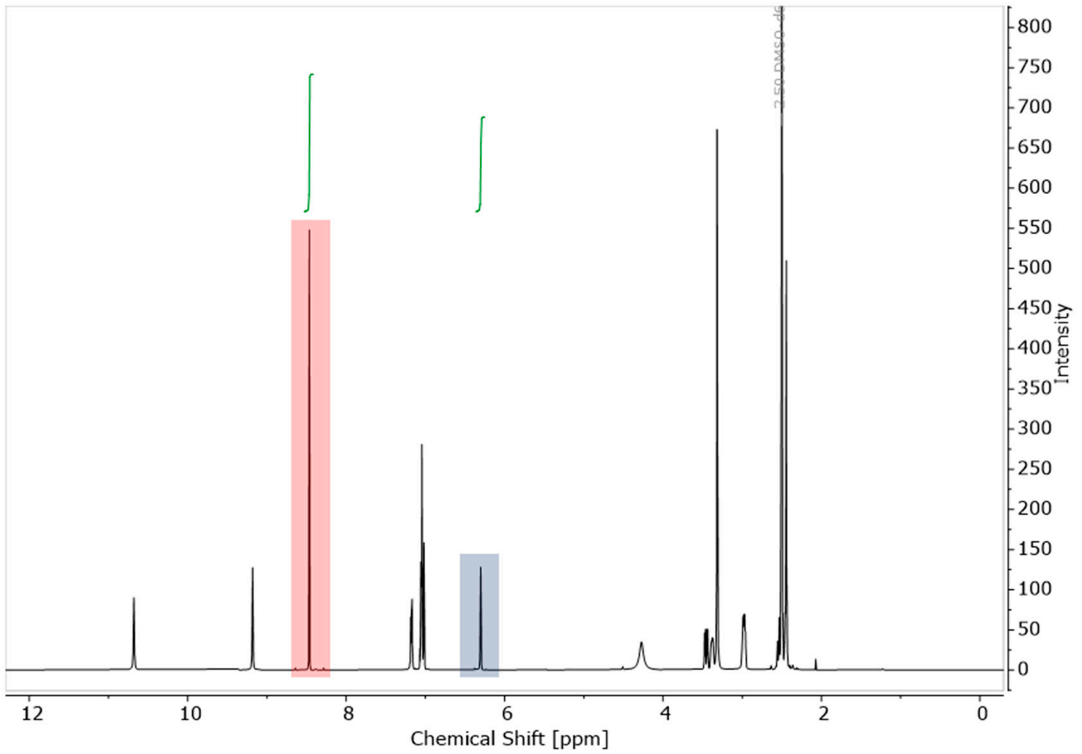

Figure S11: *q*-NMR-Spectrum for the purity assessment of (5*R*, 8*R*)-lysergic acid hydrazide. Red highlighted area at 8.47 ppm is the signal of the internal calibration standard 1,2,4,5-Tetrachloro-3-nitrobenzene. The blue area at 6.30 ppm is from the proton attached to the C9-position of the analyte, which was used for quantification.
